# Association between handgrip strength and quality of life among adults with asthma: Findings from a nationally representative Korean population

**DOI:** 10.1371/journal.pone.0338197

**Published:** 2025-12-18

**Authors:** Joo Hyeok Hong, Moon Seong Baek, Bomi Park

**Affiliations:** 1 Graduate School of Nursing and Health Professions, Chung-Ang University, Seoul, Korea; 2 Department of Internal Medicine, Chung-Ang University Hospital, Chung-Ang University College of Medicine, Seoul, Republic of Korea; 3 Department of Preventive Medicine, College of Medicine, Chung-Ang University, Seoul, Republic of Korea; Università degli Studi di Milano: Universita degli Studi di Milano, ITALY

## Abstract

Asthma is a prevalent chronic disease worldwide, and individuals with asthma often experience reduced quality of life (QOL) due to symptoms such as dyspnea and impaired physical function. Given the vulnerability of this population, identifying strategies to maintain or improve QOL is essential. Asthma-related physical inactivity and muscle weakness may increase the risk of sarcopenia, which can further deteriorate QOL. This study aimed to examine the association between handgrip strength and health-related QOL in Korean adults with asthma. We used data from the Korea National Health and Nutrition Examination Survey conducted between 2014 and 2019. A total of 867 adults aged ≥19 years who reported a physician diagnosis of asthma and completed handgrip strength and EuroQol 5-Dimension (EQ-5D) assessments were included. Participants were categorized into high- and low-handgrip-strength groups based on sex-specific thresholds (28.9 kg for men and 16.8 kg for women). The EQ-5D index was used to classify patients into low-, middle-, and high-QOL groups. Multinomial logistic regression analyses were conducted to evaluate the association between handgrip strength and QOL, adjusting for age, sex, household income, education, smoking status, high-risk alcohol use, resistance exercise, body mass index, hypertension, diabetes, arthritis, and depression. Participants with higher handgrip strength were more likely to be in the high-QOL group than those with lower handgrip strength (adjusted OR = 2.25; 95% CI: 1.22–4.16). In the subgroup analyses, the association was significant in women (adjusted OR = 2.89; 95% CI: 1.32–6.29), but not in men. A significant association was also observed in participants with as-needed medication use, but not in those with daily medication use. Higher handgrip strength was associated with better QOL in adults with asthma, particularly among women and those with less severe disease. Interventions aimed at improving muscle strength may contribute to enhanced QOL in this population.

## 1. Introduction

Asthma is one of the most common non-communicable chronic diseases, affecting over 260 million people worldwide as of 2019, and is responsible for more than 450,000 deaths annually [1]. In South Korea, the prevalence of asthma increased from approximately 1.6% in 2002 to 2.2% in 2015, with the mortality rate rising from 16.2 to 28 per 100,000 population [2]. Furthermore, recent studies have indicated a gradual increase in asthma prevalence among young adults in their 20s [3].

Asthma imposes substantial economic and social burdens because of medication costs, hospitalization, and productivity loss. For instance, in the United States, the economic burden of asthma increased from USD 56 billion in 2007 [4] to approximately USD 82 billion in 2013 [5]. In South Korea, the estimated health care cost of asthma is approximately USD 646 million [6]. Thus, asthma represents a significant public health issue not only for individuals, but also for society as a whole [7,8].

People with asthma may experience reduced physical activity owing to limitations in daily living and physical functioning, which can lead to muscle weakness and overall physical decline [9]. Prior studies have suggested that patients with chronic respiratory symptoms often find it difficult to engage in muscle-strengthening activities, which further limits their mobility and exacerbates muscle loss [10,11]. Consequently, patients with asthma may be at an increased risk of sarcopenia because of long-term inactivity [12]. Additionally, the long-term use of low-dose inhaled corticosteroids, a core element of asthma management according to the Global Initiative for Asthma (GINA) guidelines [13], has been reported to promote muscle protein breakdown and suppress synthesis, leading to muscle atrophy and reduced exercise capacity [14,15]. These effects may be particularly pronounced in patients with severe asthma undergoing prolonged treatment [16].

Asthma symptoms such as dyspnea, coughing, and chest tightness can limit participation in everyday activities, potentially resulting in impairments across occupational, academic, and recreational domains [17]. Accordingly, asthma has been associated with reduced quality of life (QOL). Inadequately controlled asthma or severe symptoms may also contribute to psychological distress, including anxiety and depression, further lowering QOL [18–22].

Previous research has shown that individuals with low handgrip strength are more likely to experience limited physical activity, difficulty with daily functioning, and psychological distress, all of which are associated with poorer QOL [23–26]. Therefore, improving muscle strength, as reflected by handgrip strength, may represent a potential intervention strategy for enhancing QOL in patients with asthma.

However, few studies have examined the epidemiological association between handgrip strength and QOL, specifically among individuals with asthma. Therefore, this study aimed to investigate the association between handgrip strength and health-related QOL (EuroQol 5-Dimension [EQ-5D]) in Korean adults with asthma and assess whether handgrip strength could serve as a potential target for interventions aimed at improving QOL in this population.

## 2. Materials and methods

### 2.1. Study population and data source

This study utilized data from the sixth (2014–2015), seventh (2016–2018), and eighth (2019) cycles of the Korea National Health and Nutrition Examination Survey (KNHANES), a nationwide cross-sectional survey conducted by the Korea Disease Control and Prevention Agency. KNHANES employs a stratified, multistage probability sampling design to collect nationally representative health data from the non-institutionalized Korean population. The survey includes a health interview, health examination, and nutrition survey. As handgrip strength was not assessed during the COVID-19 pandemic in 2020 owing to safety restrictions, only data from 2014 to 2019 were included in this study.

Adults aged ≥ 19 years who reported having ever been diagnosed with asthma by a physician and completed assessments of handgrip strength and QOL (EQ-5D) were eligible. Asthma severity was classified based on self-reported medication use in the past year with reference to the 2021 GINA guidelines. Participants were categorized into two medication-use patterns: as-needed medication and daily medication, based on their self-reported frequency of asthma medication use during the past year [27].

Participants were excluded if they were under 19 years of age, had a history of cancer (to minimize bias from serious comorbid conditions), or had missing data for any of the key variables, including EQ-5D, body mass index (BMI), hypertension, diabetes, household income, smoking status, education level, high-risk alcohol use, or resistance exercise.

The study protocol was approved by the Institutional Review Board of Chung-Ang University (IRB No. 1041078-20241018-HR-295). The requirement for informed consent was waived because the analysis was conducted using de-identified, publicly available data. The data were accessed for research purposes between November 15, 2024, and December 31, 2024, and the authors did not have access to any information that could identify individual participants during or after data collection.

### 2.2. Handgrip strength

In this study, handgrip strength was used as an indirect marker (proxy) of sarcopenia, as data on muscle mass and physical performance tests were not available in the KNHANES. Handgrip strength was measured using a digital dynamometer (T.K.K 5401, Japan) in accordance with the KNHANES protocols. Each participant performed three trials per hand, and the highest value among the six measurements was used for the analysis.

Handgrip strength was categorized as low (<28.9 kg for men and <16.8 kg for women) or high, based on Korean population-specific reference data derived from a nationally representative sample [28]. These cutoffs were chosen to maintain comparability with other Korean studies. Although they differ slightly from the Asian Working Group for Sarcopenia (AWGS) recommendations (<28 kg for men and <18 kg for women), the Korean reference values were considered more appropriate given population-specific anthropometric characteristics and measurement protocols.

In addition, handgrip strength was analyzed as a continuous variable (per 1-kg and per 1-SD increase). Linearity of the association was assessed by comparing linear and restricted cubic spline (3-knot) models using the Akaike Information Criterion (AIC). A ΔAIC < 2 indicated no meaningful improvement in model fit by the spline, supporting a linear specification.

### 2.3. Quality of life (EQ-5D)

Health-related QOL was assessed using the EQ-5D questionnaire, a standardized instrument that measures five dimensions: mobility, self-care, usual activities, pain/discomfort, and anxiety/depression [29,30]. Each item is rated on a three-point scale (1 = no problems, 2 = some problems, 3 = extreme problems), and the responses are converted to a single index value [31]. Based on the distribution of the index scores, the participants were categorized into three QOL groups:

1. High QOL: EQ-5D index = 1.0
2. Middle QOL: EQ-5D index < 1.0 and ≥ median value among those not in the high QOL group
3. Low QOL: EQ-5D index < median value

### 2.4. Covariates

Covariates were selected based on previous literature linking them to handgrip strength and/or QOL [32–39]. These included age, sex, household income (categorized into tertiles: low, middle, and high), education level (middle school or less vs. high school or more), smoking status (current smoker vs. former/never smoker), high-risk alcohol use (defined as ≥7 drinks per occasion for men or ≥5 drinks per occasion for women, at least twice per week), resistance exercise (≥2 days per week vs. < 2 days), BMI (categorized as normal <23, overweight 23–24.9, and obese ≥25), and physician-diagnosed hypertension, diabetes, arthritis, and depression.

### 2.5. Statistical analysis

Descriptive statistics were used to summarize the demographic and clinical characteristics of the study population. Participants with missing values for any variable included in the model were excluded from the respective analyses, without statistical imputation. Categorical variables are presented as unweighted frequencies and weighted percentages, and continuous variables are presented as medians and interquartile ranges (IQRs). Chi-squared tests were used to examine the associations between categorical variables, and Wilcoxon rank-sum tests were used to compare continuous variables.

Multinomial logistic regression analyses were conducted to examine the association between handgrip strength and EQ-5D QOL. Analyses were stratified by sex and asthma medication-use pattern. Because KNHANES uses a complex sampling design, all analyses incorporated survey weights, strata, and primary sampling units using the SURVEY procedures in SAS version 9.4 (SAS Institute Inc., Cary, NC, USA). The results are presented as odds ratios (ORs) and 95% confidence intervals (CIs), with statistical significance defined as a two-sided p-value < 0.05.

## 3. Results

**Fig 1:**
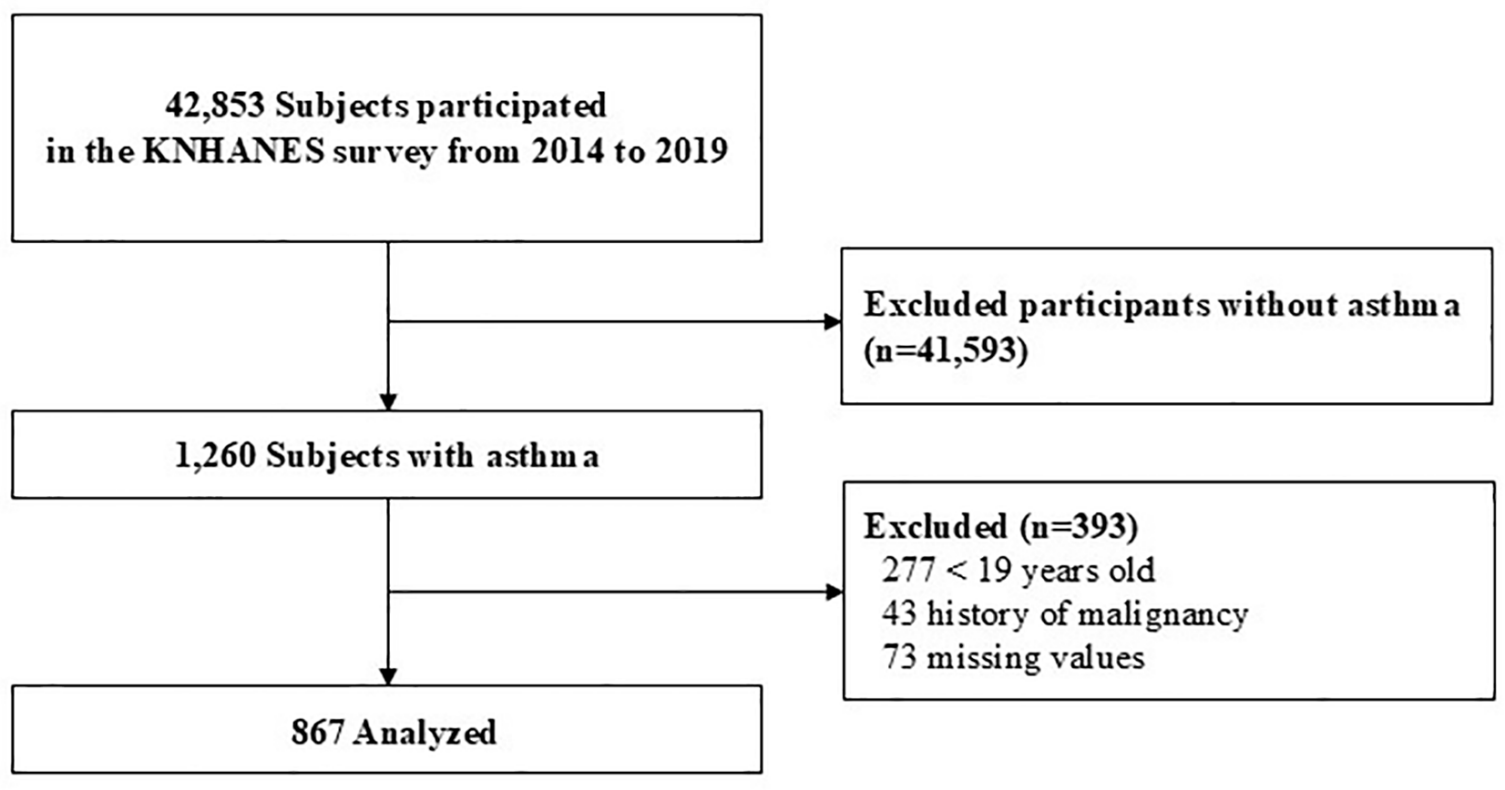
Flow diagram of participant selection from the KNHANES.

Among the 42,853 participants in the 2014–2019 KNHANES, 1,260 individuals reported a physician diagnosis of asthma. After excluding those under 19 years of age, those with a history of cancer (n = 43), and individuals with missing values in key variables (n = 73), a total of 867 participants were included in the final analysis (Fig 1). 

Table 1 summarizes the demographic characteristics of the participants by sex and asthma medication-use pattern. Of the 867 participants, 341 (39.3%) were men and 526 (60.7%) were women. A total of 678 (78.2%) patients reported as-needed medication use, and 189 (21.8%) reported daily medication use. Based on the handgrip strength criteria, 729 (84.1%) participants were classified in the high-handgrip group and 138 (15.9%) in the low-handgrip group. A higher proportion of men (86.8%) belonged to the high-handgrip-strength group compared with women (82.3%; p = 0.072). Additionally, the proportion of participants with high handgrip strength was greater among those with as-needed medication (86.4%) than among those with daily medication (75.7%) (p < 0.001). A total of 474 (54.7%) participants were classified into the high QOL group, and 160 (18.4%) into the low QOL group. Among men, 64.5% were in the high group and 13.2% in the low group; among women, 48.3% were in the high group and 21.9% in the low group. In participants with as-needed medication, 58.4% were classified in the high group and 16.1% in the low group, whereas in those with daily medication, 41.3% were in the high group and 27.0% in the low group.

**Table 1:** Demographic and clinical characteristics of study participants by sex and asthma medication-use pattern.

Variables	Total (N = 867)	By sex			By asthma medication-use pattern		
		Men (N = 341)	Women (N = 526)	*p-value*	As-needed medication (N = 678)	Daily medication (N = 189)	*p-value*
**Age**	52.7 (36.0–70.0)	46.0 (29.0–70.0)	58 (40.0–70.0)	<0.001	50.0 (33.0–66.0)	69.0 (55.0–76.0)	<0.001
**Sex**							0.48
** Male**	341 (39.3)	–	–		267 (39.4)	74 (39.2)	
** Female**	526 (60.7)	–	–		411 (60.6)	115 (60.8)	
**BMI**				0.02			0.12
** Normal**	328 (37.8)	113 (33.1)	215 (40.9)		261 (38.5)	67 (35.5)	
** Overweight**	201 (23.2)	88 (25.8)	113 (21.5)		158 (23.3)	43 (22.7)	
** Obese**	338 (39.0)	140 (41.1)	198 (37.6)		259 (38.2)	79 (41.8)	
**HTN**				<0.001			<0.001
** No**	355 (41.0)	131 (38.4)	224 (42.6)		311 (45.9)	44 (23.3)	
** Pre-HTN**	205 (23.6)	103 (30.2)	102 (19.4)		162 (23.9)	43 (22.7)	
** Yes**	307 (35.4)	107 (31.4)	200 (38.0)		205 (30.2)	102 (54.0)	
**Diabetes**				0.02			<0.001
** No**	404 (46.6)	175 (51.3)	229 (43.5)		344 (50.8)	60 (31.7)	
** Pre-DM**	324 (37.4)	112 (32.9)	212 (40.3)		243 (35.8)	81 (42.9)	
** Yes**	139 (16.0)	54 (15.8)	85 (16.2)		91 (13.4)	48 (25.4)	
**Household income**				0.01			<0.001
** Low**	237 (27.3)	79 (23.2)	158 (30.0)		153 (22.5)	84 (44.4)	
** Middle**	410 (47.3)	166 (48.7)	244 (46.4)		332 (49.0)	78 (41.3)	
** High**	220 (25.4)	96 (28.1)	124 (23.6)		193 (28.5)	27 (14.3)	
**Smoking status**				<0.001			0.06
** No**	719 (82.9)	236 (69.2)	483 (91.8)		554 (81.7)	165 (87.3)	
** Yes**	148 (17.1)	105 (30.8)	43 (8.2)		124 (18.3)	24 (12.7)	
**Educational level**				<0.001			<0.001
** ≤ Middle school**	352 (40.6)	96 (28.1)	256 (48.7)		242 (35.7)	110 (58.2)	
** ≥ High school**	515 (59.4)	245 (71.9)	270 (51.3)		436 (64.3)	79 (41.8)	
**High-risk alcohol drinking**				<0.001			0.47
** No**	784 (90.4)	286 (83.9)	498 (94.7)		605 (89.2)	179 (94.7)	
** Yes**	83 (9.6)	55 (16.1)	28 (5.3)		73 (10.8)	10 (5.3)	
**Resistance exercise**				<0.001			0.01
** ≤ 1 day/week**	684 (78.9)	236 (69.2)	448 (85.2)		520 (76.7)	164 (86.8)	
** ≥ 2 days/week**	183 (21.1)	105 (30.8)	78 (14.8)		158 (23.3)	25 (13.2)	
**Arthritis**				<0.001			<0.001
** No**	660 (76.1)	312 (91.5)	348 (66.2)		538 (79.4)	122 (64.6)	
** Yes**	207 (23.9)	29 (8.5)	178 (33.8)		140 (20.6)	67 (35.4)	
**Depression**				<0.001			0.15
** No**	784 (90.4)	326 (95.6)	458 (87.1)		618 (91.1)	166 (87.8)	
** Yes**	83 (9.6)	15 (4.4)	68 (12.9)		60 (8.9)	23 (12.2)	
**Handgrip**				0.07			<0.001
** High**	729 (84.1)	296 (86.8)	433 (82.3)		586 (86.4)	143 (75.7)	
** Low**	138 (15.9)	45 (13.2)	93 (17.7)		92 (13.6)	46 (24.3)	
**EQ5D index**				<0.001			<0.001
** High**	474 (54.7)	220 (64.5)	254 (48.3)		396 (58.4)	78 (41.3)	
** Intermediate**	233 (26.9)	76 (22.3)	157 (29.8)		173 (25.5)	60 (31.8)	
** Low**	160 (18.4)	45 (13.2)	115 (21.9)		109 (16.1)	51 (27.0)	

Values are presented as median (Q1-Q3) or unweighted frequency (weighted %).

BMI, body mass index; HTN, hypertension.

Table 2 shows the association between handgrip strength and EQ-5D. After adjusting for covariates, participants in the high-handgrip-strength group had higher odds of belonging to the high-QOL group than those in the low-handgrip-strength group (adjusted OR = 2.25; 95% CI: 1.22–4.16). The odds of being in the middle-QOL group were also higher (adjusted OR = 1.68; 95% CI: 0.92–3.04), although the result was not statistically significant.

**Table 2:** Multinomial logistic regression of categorical handgrip strength and EQ-5D.

	EQ5D index categories^d^		
Model^a^	Low group^b^	Intermediate group	High group
**Unadjusted model**	Ref	3.09 (1.81–5.28)	7.85 (4.68–13.18)
**Adjusted model** ^**c**^	Ref	1.68 (0.92–3.04)	2.25 (1.22–4.16)

^a^All regression analyses were conducted using weights to account for the complex sampling design of the Korean National Health and Nutrition Examination Survey (KNHANES).

^b^The low-EQ5D group was used as the reference category in the multinomial logistic regression analysis.

^c^Multinomial logistic regression analysis was adjusted for confounders, including age, sex, BMI, hypertension, diabetes, income, smoking status, education level, high-risk drinking rates, resistance exercise, arthritis, and depression.

^d^Odds ratios (ORs) with 95% confidence intervals (CIs) are presented.

Table 3 presents the results of the stratified analysis by sex. Among men, high handgrip strength was associated with increased odds of being in the middle-QOL group (OR = 1.59; 95% CI: 0.48–5.28) and the high-QOL group (OR = 1.75; 95% CI: 0.56–5.49); however, these associations were not statistically significant. In contrast, among women, those in the high-handgrip-strength group had significantly higher odds of being in the high-QOL group (OR = 2.89; 95% CI: 1.32–6.29). The odds for the middle-QOL group were also elevated (OR = 1.93; 95% CI: 0.96–3.86), though they were not statistically significant.

**Table 3:** Multinomial logistic regression of categorical handgrip strength and EQ-5D by sex.

	EQ5D index categories^d^		
Model^a^	Low group^b^	Intermediate group	High group
**Men**			
** Unadjusted model**	Ref	1.92 (0.64–5.78)	5.95 (2.24–15.82)
** Adjusted model** ^**c**^	Ref	1.59 (0.48–5.28)	1.75 (0.56–5.49)
**Women**			
** Unadjusted model**	Ref	3.89 (2.19–6.92)	8.31 (4.41–15.65)
** Adjusted model** ^**c**^	Ref	1.93 (0.96–3.86)	2.89 (1.32–6.29)

^a^All regression analyses were conducted using weights to account for the complex sampling design of the Korean National Health and Nutrition Examination Survey (KNHANES).

^b^The low-EQ5D group was used as the reference category in the multinomial logistic regression analysis.

^c^Multinomial logistic regression analysis was adjusted for confounders, including age, BMI, hypertension, diabetes, income, smoking status, education level, high-risk drinking rates, resistance exercise, arthritis, and depression.

^d^Odds ratios (ORs) with 95% confidence intervals (CIs) are presented.

Table 4 shows the results stratified by asthma medication-use pattern. Among the as-needed medication group, those with high handgrip strength had significantly higher odds of being in the high-QOL group (OR = 2.36; 95% CI: 1.12–4.99) and moderately higher odds for the middle-QOL group (OR = 1.92; 95% CI: 0.92–3.98), though the latter did not reach statistical significance. Among the daily medication group, the associations between handgrip strength and QOL were not statistically significant (OR = 1.83; 95% CI: 0.58–5.79).

**Table 4:** Multinomial logistic regression of categorical handgrip strength and EQ-5D by asthma medication-use pattern.

		EQ5D index categories^d^	
Model^a^	Low group^b^ (Reference)	Intermediate group OR (95% CI)	High group OR (95% CI)
**As-needed medication**			
** Unadjusted model**	1.00	4.21 (2.09–8.50)	9.40 (4.73–18.67)
** Adjusted model** ^**c**^	1.00	1.92 (0.92–3.98)	2.36 (1.12–4.99)
**Daily medication**			
** Unadjusted model**	1.00	2.38 (0.83–6.88)	4.40 (1.51–12.81)
** Adjusted model** ^**c**^	1.00	1.11 (0.35–3.52)	1.83 (0.58–5.79)

^a^All regression analyses were conducted using weights to account for the complex sampling design of the Korean National Health and Nutrition Examination Survey (KNHANES).

^b^The low-EQ5D group was used as the reference category in the multinomial logistic regression analysis.

^c^Multinomial logistic regression analysis was adjusted for confounders, including age, sex, BMI, hypertension, diabetes, income, smoking status, education level, high-risk drinking rates, resistance exercise, arthritis, and depression.

^d^Odds ratios (ORs) with 95% confidence intervals (CIs) are presented.

Table 5 presents the associations between continuous handgrip strength and EQ-5D. A significant positive association was observed among women, whereas no significant association was found in other subgroups. Comparison between restricted cubic spline and linear models yielded ΔAIC < 2, indicating no evidence of nonlinearity. The direction and pattern of the associations were consistent with those observed in the categorical analyses, showing a higher QOL with greater handgrip strength. However, the effect sizes were smaller in the continuous models, as they represent the change per unit increase rather than contrasts between high and low categories.

**Table 5:** Multinomial logistic regression of continuous handgrip strength (per kg and per SD) and EQ-5D.

	EQ5D index categories^d^		
Model^a, b^	Low group^c^	Intermediate group	High group
**Per 1 kg increase**			
** Total**	Ref	0.99 (0.95–1.03)	1.01 (0.97–1.05)
** Sex**			
** Men**	Ref	1.00 (0.94–1.07)	1.00 (0.94–1.07)
** Women**	Ref	1.06 (0.99–1.14)	1.09 (1.02–1.17)
** Medication-use pattern**			
** As-needed medication**	Ref	0.99 (0.93–1.05)	1.02 (0.97–1.08)
** Daily medication**	Ref	0.96 (0.91–1.06)	0.97 (0.90–1.02)
**Per 1 SD increase**			
** Total**	Ref	0.90 (0.59–1.37)	1.13 (0.74–1.72)
** Sex**			
** Men**	Ref	1.03 (0.60–1.78)	1.03 (0.62–1.71)
** Women**	Ref	1.38 (0.95–2.00)	1.59 (1.09–2.31)
** Medication-use pattern**			
** As-needed medication**	Ref	0.89 (0.49–1.60)	1.27 (0.71–2.28)
** Daily medication**	Ref	0.75 (0.42–1.34)	0.68 (0.39–1.18)

^a^All regression analyses were conducted using weights to account for the complex sampling design of the Korean National Health and Nutrition Examination Survey (KNHANES).

^b^Models were adjusted for confounders, including age, sex, BMI, hypertension, diabetes, income, smoking status, education level, high-risk drinking rates, resistance exercise, arthritis, and depression. Sex was not included as a covariate in sex-stratified analyses.

^c^The low-EQ5D group was used as the reference category in the multinomial logistic regression analysis.

^d^Odds ratios (ORs) with 95% confidence intervals (CIs) are presented.

## 4. Discussion

This study investigated the association between handgrip strength and health-related quality of life (EQ-5D) in Korean adults with asthma. Individuals with higher handgrip strength were significantly more likely to report better quality of life, particularly women and those using as-needed asthma medication. These findings suggest that maintaining or improving muscular strength may play an important role in enhancing the quality of life among patients with asthma.

When handgrip strength was analyzed as a continuous variable, the direction of the association with EQ-5D was consistent with that observed in the categorical analyses, showing a higher QOL with increasing grip strength. Although the magnitude of the association was smaller and statistical significance was observed only among women, the overall pattern remained linear and positive.

Handgrip strength was used in this study as a proxy for sarcopenia. Previous studies have established strong correlations between handgrip strength and muscle mass and function, making it a reliable marker of physical capability [40–42]. Prior studies have shown that reduced handgrip strength is associated with lower QOL in specific populations, such as older adults [43] and patients with arthritis [24], chronic obstructive pulmonary disease (COPD) [44], and osteoporosis [45]. However, to the best of our knowledge, no previous study has examined this relationship in patients with asthma. Our findings expand on the literature by showing that lower handgrip strength is also associated with lower QOL in adults with asthma. While earlier studies have reported associations between low handgrip strength and increased mortality in respiratory diseases [46] and severe airflow limitation [47], our findings demonstrated a novel association between handgrip strength and QOL in this population.

Stratified analyses revealed that the association between handgrip strength and QOL was significant only in women. This may be because of sex-specific differences in muscle mass and the prevalence of sarcopenia. Research has shown that sarcopenia is more prevalent in women than in men as age increases [48], possibly because of hormonal factors. Testosterone levels in men rise sharply after puberty, leading to greater muscle mass and strength than in women of the same age and training level [49,50]. Men also tend to have a larger cross-sectional area of muscle and slower rates of muscle loss with age [32]. In contrast, postmenopausal women experience a decline in estrogen levels, which contributes to reduced muscle mass and joint pain in nearly 77% of women [33]. Studies have also reported that women with low handgrip strength are significantly more likely to experience mobility issues (OR = 2.12) and limitations in daily activities (OR = 2.04) [34].

Moreover, women may experience greater psychological benefits from physical improvement than men. Increased muscle strength in women has been linked to reduced stress and improved emotional stability [35,36], and women are generally more prone to depression and anxiety, which may further influence their QOL [37].

Interestingly, the association between handgrip strength and QOL was significant only in participants with as-needed medication use. This finding is consistent with those of previous studies on patients with respiratory disease, where those with milder disease severity showed stronger associations between physical function and QOL [38]. One possible explanation is that patients with severe chronic diseases, such as advanced COPD, may experience limited QOL improvements despite treatment, as disease progression often outweighs the benefits of physical improvement [39]. Although the exact mechanisms remain unclear, these findings suggest that physical interventions may be more effective in improving the QOL of patients with less severe asthma.

Several biological mechanisms may underlie the reduced muscle strength observed in adults with asthma–chronic systemic inflammation (IL-6, TNF-α) [51,52], intermittent hypoxia with ventilatory limitation during exacerbations [53], physical deconditioning driven by fear of dyspnea [53], and steroid-induced myopathy with prolonged corticosteroid exposure [54,55]. These processes can reduce skeletal-muscle function and exercise capacity, leading to mobility limitations, greater fatigue, and lower performance of daily activities, which are reflected in EQ-5D domains; this pathway provides biological plausibility for the observed association between lower handgrip strength and lower quality of life in asthma.

Overall, this study has several important public health and clinical implications. First, asthma is associated with substantial reductions in QOL owing to both physical and psychological burdens. Previous studies have shown that QOL decreases as asthma severity increases [56,57], and even with adequate treatment, many patients with asthma report lower QOL than patients without asthma [58] . Therefore, improving the QOL of patients with asthma remains a crucial goal. Our findings indicate that interventions to improve muscle strength, particularly handgrip strength, may be a promising strategy to support better health outcomes, especially for women and individuals with mild asthma. Rehabilitation programs or physical activity interventions designed to enhance muscular strength can be integrated into routine asthma care.

To our knowledge, this is one of the few studies to explore the relationship between handgrip strength and QOL in patients with asthma using nationally representative data. Stratified analysis by sex and asthma severity provided additional insights into the subgroups that may benefit most from strength-based interventions. These findings may support the development of tailored strategies to manage asthma and enhance patient-centered outcomes.

However, this study has some limitations. First, its cross-sectional design precluded the determination of a causal relationship between handgrip strength and QOL. Second, although multiple covariates were adjusted for, the potential for residual confounding variables remains because some were unmeasured. Third, because the study population was limited to Korean adults, the generalizability of the findings to other ethnic or cultural groups may be limited. Future longitudinal and interventional studies are needed to confirm whether improving handgrip strength leads to improvements in QOL and to expand on these findings to broader populations. Fourth, EQ-5D was analyzed as a categorical variable (low, intermediate, and high) rather than as a continuous score. Although this approach was chosen to address the marked right-skew and ceiling effect commonly observed in EQ-5D data, it may have resulted in a loss of statistical power and information compared with treating EQ-5D as a continuous variable. Finally, the distinction between as-needed medication and daily medication was based on the self-reported frequency of use. While practical, this measure does not fully reflect the GINA-defined multidimensional severity and may involve misclassification due to differences in adherence.

## 5. Conclusion

This study demonstrated a significant association between handgrip strength and QOL (EQ-5D) in Korean adults with asthma. Individuals with higher handgrip strength were more likely to report a better QOL, particularly women and those with mild asthma severity. These findings highlight the potential value of incorporating muscle-strengthening interventions into asthma management strategies to improve patient well-being. Strengthening programs targeting specific subgroups, such as women and those with less severe disease, may be especially effective. Future longitudinal and intervention-based research is needed to further explore causal relationships and assess the impact of muscular strength improvements on QOL in diverse asthma populations.
